# Medical undergraduates’ perceptions on the end of course assessment in Surgery in a developing country in South Asia

**DOI:** 10.1186/s13104-018-3828-1

**Published:** 2018-10-16

**Authors:** Savindi De Mel, Umesh Jayarajah, Sanjeewa A. Seneviratne

**Affiliations:** Department of Surgery, Faculty of Medicine, University of Colombo, No. 25, Kynsey Road, Colombo 08, Sri Lanka

**Keywords:** Assessment, Feedback, Medical education, Undergraduate, Surgery

## Abstract

**Objective:**

This study reports findings from a feedback assessment conducted among final year medical undergraduates on the end of course assessment in Surgery. A self-administered questionnaire was used among 201 final year medical undergraduates of the Faculty of Medicine Colombo to collect students’ perceptions on clinical assessment (i.e. long and short cases), performance of examiners during clinical assessments and student perceptions on different types of undergraduate assessments in Surgery.

**Results:**

Approximately 90% of undergraduates perceived that both long and short case assessments were fair in assessing their knowledge and clinical skills. On the overall assessment in Surgery, approximately 90% agreed that tasks reflected those taught, assessment covered a wide area of knowledge and skills in Surgery and time given for assessment was adequate. Most felt long case to be the best method in assessing whether one is a safe doctor with good communication skills and ability to apply knowledge practically. Thus, a majority of students were satisfied with the current assessment system and most perceived the clinical component to be superior to all other components in assessing whether a student is suitable to become a good and a safe doctor.

**Electronic supplementary material:**

The online version of this article (10.1186/s13104-018-3828-1) contains supplementary material, which is available to authorized users.

## Introduction

It is well established that in higher education, assessments define the curriculum, at least from the students’ point of view [1]. Accurate measurement of core competencies in medical knowledge is an essential component for evaluation in clinical medicine [2]. Furthermore, students perceive it as the most powerful driver determining what and how they learn [3]. In medical education, well established models have shown that actively involving the learner in the feedback process, identifying areas for development, and formulating a mutually agreed action plan are crucial to maximise the effectiveness training and assessment [4].

Assessments of clinical competence, where decisions are made to decide whether an undergraduate is suitable to become a medical practitioner include assessments of competence in domains of knowledge, skills and attitudes. These assessments need to be aligned with key assessment principles including validity, reliability, and standard setting, as well as clarity about their function [5]. Multiple choice questions (MCQ), structured essays and oral examinations could be used to test factual recall and applied knowledge, but more sophisticated methods are needed to assess clinical performance where skills and attitudes need be assessed beyond factual recall [5]. Directly observed long and short cases, objective structured clinical examinations (OSCE), and standardised patients are frequently used to assess competence in clinical performance and communication skills. The goal of assessment in medical education remains the development of reliable measurements of student performance which has a predictive value determining subsequent clinical competence and performance as a medical practitioner [6].

Medical undergraduates at Faculty of Medicine, University of Colombo, which is the second oldest medical school in South Asia established in 1870, undergo a 5 year medical curriculum comprising of five different streams [7]. At the end of 5 years, a final examination is held which includes five clinical subjects; Medicine, Surgery, Paediatrics, Obstetrics and Gynaecology and Psychiatry. Final assessment in Surgery comprises of multiple types of assessments including both clinical and written components. The written component consisted of MCQs and SEQs. MCQs consisted of both true/false type questions and single best response type questions which were targeted to assess the basic sciences related to Surgery, diagnostic thinking and application of knowledge, evaluating the examinee’s ability to integrate, synthesize, and judge medical information. SEQs were based on clinical problems and requires the students to provide structures answers on investigations, diagnosis and management of common surgical conditions and emergencies.

The clinical skills assessment consisted mainly on short and long cases. In short cases, the candidate is given 20 min to examine real patients with common surgical conditions with or without brief history taking. The ability of the candidate to perform a relevant examination, and interpretation of findings are assessed with a general discussion. In long cases, candidates are given 40 min for detailed assessment of a real patient with a surgical condition and a 20 min interview with examiners to discuss about the clinical findings and management of the given patient.

Viva or oral examination include two 10 min stations to assess students’ knowledge on surgical procedures, emergencies and basic anaesthesiology.

OSCE comprising of 10 stations, is a timed, multi-station examination in which learners perform tasks such as interviews, physical exams, clinical/resuscitative procedures and counselling in realistic settings. At each station learner performance is evaluated with specific checklists and global rating scales. OSCE’s enable the same clinical scenarios to be presented to many candidates. Both OSCE and a viva examination are conducted as continuous assessments during the final year clinical rotation, and marks from these assessments also contribute to the final mark in Surgery. An adequate competence (score of ≥ 50%) in both theory and clinical components are required for a ‘pass’ in Surgery, as similar with other clinical subjects.

The present study reports the findings from a feedback assessment conducted among final year students on the end of course assessment in Surgery at the Faculty of Medicine, Colombo, Sri Lanka.

## Main text

### Methods

#### Study population

A feedback assessment on the end of course assessment in Surgery was done among undergraduates of the Faculty of Medicine, University of Colombo, Sri Lanka who underwent the final examination in December 2016. Ethical clearance was obtained from the Ethics Review Committee, Faculty of Medicine, University of Colombo.

#### Study instrument

A self-administered, anonymised questionnaire (Additional file 1) was given to all undergraduates immediately after the completion of the final component of the end of course assessment in Surgery. A 100% response rate was achieved by instituting the questionnaire to each student immediately after the completion of the clinical assessment, before they left the examination hall. This was done not only to assure 100% response rate, but also to minimize recall bias. The questionnaire included questions regarding students’ perceptions on the performance of examiners during clinical component of the assessment (i.e. long and short cases), perceptions on clinical assessment and perceptions on overall undergraduate assessment in Surgery. Likert scales were used to assess whether students agree or disagree with the statements included in the questionnaire.

The questionnaire was inclusive of questions regarding each format of the Surgery examination, namely MCQ, SEQ, viva, OSCE and clinical assessment. Perceptions as to which of the above assesses practical application of clinical knowledge, identify whether one is a good and safe doctor, test communication and counselling skills, demands organizational and time management skills and tests overall quality of performance were included in the questionnaire.

### Results

All 201 (100%) students who underwent the final year assessment in Surgery completed the questionnaire. For all questions the response rates were over 96% with most questions having a response rate of > 98%.

**Fig. 1:**
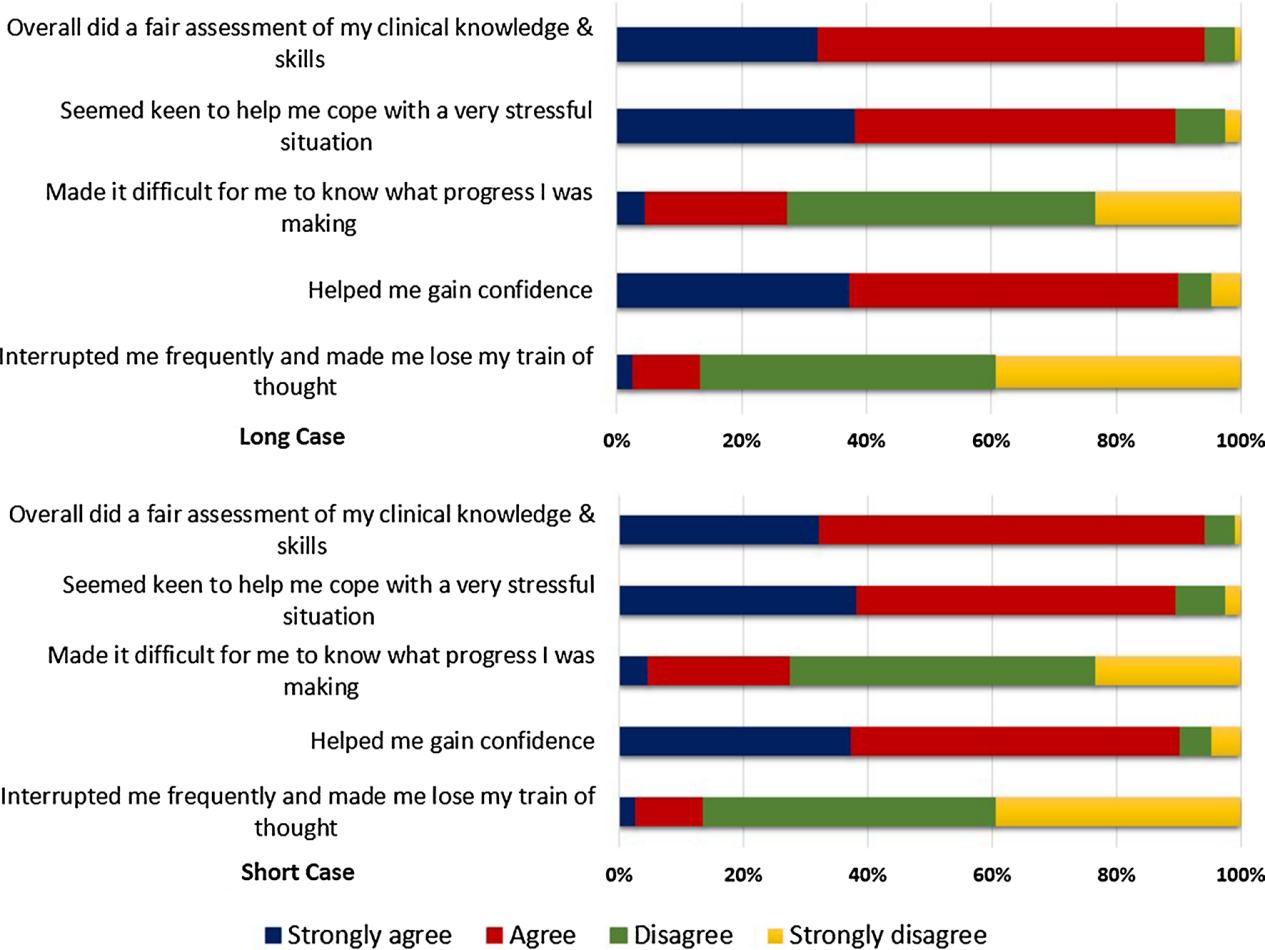
Undergraduates’ perception on conduct and performance at the long and short clinical case of Surgery assessment

Approximately 90% of undergraduates thought that both long and short case assessments were fair in assessing their knowledge and skills (Fig. 1). Assessment of other components of examiner conduct was almost identical between long and short clinical cases. For instance, students were in general agreement that examiners were helping the students deal with a very stressful situation (84.5% and 86.8%), were indicating the progress students were making during the assessment (73.4% and 72.5%), were helping students gain confidence (87.5% and 90.1%) with minimum interruptions which could interfere with their trains of thought (86.1% and 86.8%) during long and short cases, respectively.

**Fig. 2:**
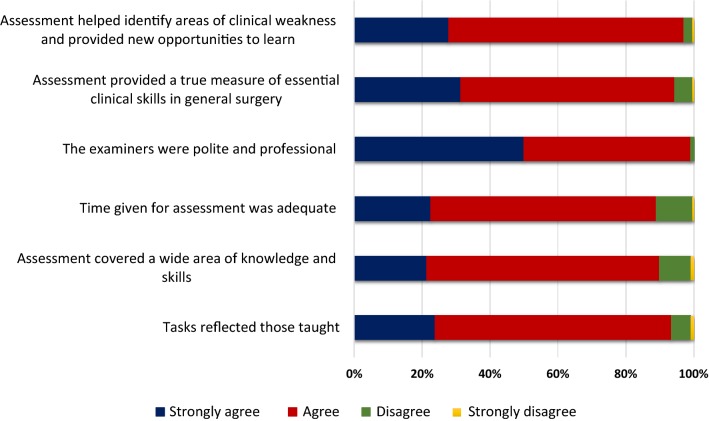
Undergraduates’ overall perception on the clinical assessment in Surgery

With regard to overall clinical assessment in Surgery (Fig. 2), 93.4% agreed that tasks reflected those taught and 88.5% agreed that assessment covered a wide area of knowledge and skills in Surgery. Almost 90% agreed that time given for assessment was adequate, 99% agreed that examiners were polite and professional and 95% agreed that assessment provided a true measure of essential clinical skills in General Surgery. The rate of agreement that clinical assessment helped them identify areas of clinical weakness and provided new opportunities to learn was 97%.

**Fig. 3:**
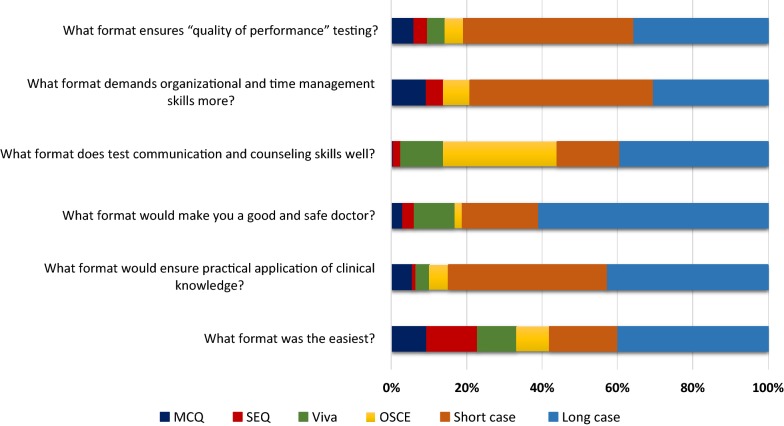
Undergraduates’ perception on different types of assessments included in Surgery assessment

Final segment of the questionnaire analysed students’ perceptions on different components of the Surgery assessment (Fig. 3). For most components of the questionnaire, which include areas such as the format that assess whether one is a safe doctor, practical application of knowledge and communication skills testing, students’ felt that long clinical case was the best with rates varying between 40 and 60%. Short cases scored highest rates for quality of performance testing and time management skills with rates of 45.2% and 48.7%, respectively. In rest of the areas assessed short cases scored second behind long case. Interestingly, almost 40% felt that long case was the easiest to pass out of all different types of assessments.

Thirty percent felt that OSCE was the best format to assess communication skills. Scores for MCQ, SEQ and viva in every component was < 10%, except that 15% thought SEQ was the easiest component of the Surgery assessment.

### Discussion

The study which was conducted to find out student perceptions on the end of course assessment in Surgery has shown that a majority of students are satisfied with the current assessment system as being fair in relation to the way it is conducted and content that is assessed. Further, most undergraduates perceived that clinical component is by far superior to all other components in assessing whether a student is suitable to become a good and safe doctor.

Conducting proper assessments in a medical curriculum is a difficult task and have multiple purposes [8]. First, it helps decide which undergraduates are fit enough to be released into the community potentially to work as independent medical practitioners [9]. Second, assessments drive student learning and it communicates to students what teachers want them to learn [6, 10]. Therefore, it is essential that the assessment system is in line with the expected outcomes and objectives of the medical curriculum. Further, especially in medical education, assessments that are in higher stages of Miller’s pyramid are encouraged over assessments of theory knowledge only [11].

An assessment should be properly planned and carried out to ensure that it assesses expected outcomes of the curriculum. Regardless, success at an assessment does not necessarily mean that a student is fully competent [5, 9]. Ideally, the assessment of competence should provide insight into actual performance, as well as the capacity to adapt to change, and find and generate new knowledge [5, 6]. Of the assessment types included in the current Surgery assessment, long case probably was the most suitable, followed by the short cases in achieving many of these competencies, which is reflected in student feedback.

Although clinical assessments in the form of long and short cases are relatively good tools in the assessment of clinical competence, there are many drawbacks [12]. Heterogeneity of patients and inter-examiner variability and examiner bias make it extremely hard to standardize the clinical assessment [13–15]. Hence there is a trend towards conducting clinical examiners in an OSCE format with simulated patients and standardized marking schemes, which helps to reduce such heterogeneity [16, 17]. Although a significant majority perceived that examiners of the present assessment were generally helpful and were not interfering with students’ thought process, just over 10% felt that they were not helpful or were unable to perform a fair assessment of the candidate. This aspect of the examination needs to be looked at in further detail and necessary remedial action instituted to ensure such biases are minimized.

Faculty of Medicine, Colombo currently is in a process of re-evaluating the assessments in Surgery to enhance validity and reliability, and to ensure expected outcomes of the curriculum are assessed adequately and appropriately. In this regard, several changes have been proposed and are being evaluated at present. Proposed changes include introduction of observed history taking and examination in long case to be done by two separate examiner panels and, introduction of four separate bays for short cases covering different body regions to be performed by four different examiner panels. These changes would ensure a more comprehensive assessment of clinical skills, and involvement of a greater number of examiners would help reduce examiner bias. However, from a logistical point of view these changes would pose several challenges. For instance, for clinical assessment alone, each student will go through twelve examiners compared with the four that is required at present, significantly increasing the number of examiners required to conduct the examination.

This study is the first of its kind from the Faculty of Medicine, Colombo where a feedback has been used to assess student perceptions on the end of course assessment in Surgery. Several important themes have emerged which potentially could help introduce changes to the present system of assessment. Further studies looking at the student perceptions on other aspects of the curriculum and a repeat assessment once the proposed changes are introduced will help to improve the present curriculum.

### Conclusion

A majority of students were satisfied with the current assessment system as being fair in relation to the way it is conducted and content that is assessed. Most perceived the clinical component to be superior to all other components in assessing whether a student is suitable to become a good and a safe doctor.

## Limitations

As feedback was obtained from all students who completed the assessment, findings of this study are representative of the batch and is the main strength of the present study. However, we have identified several limitations. First, the feedback was limited to a few key areas only. Hence many other areas including specific reasons for perceptions and whether these were related to specific examiners or students were not studied. Further, we did not assess whether there were correlations among feedback scores and student performance or behaviours of examiners. As the study included only a single batch from a single faculty, findings may not be generalizable to other batches or students from other faculties in Sri Lanka. The absence of a qualitative component in the assessment is a major limitation. However, this quantitative assessment will be helpful as a preliminary study to recognize the relative importance of the areas to be included in further qualitative assessments.

## Additional file

**Additional file 1.** Questionnaire used in the survey. The questionnaire used in this study is a quantitative assessment of students’ perception on end of course assessment in Surgery. The questionnaire includes questions regarding students’ perceptions on the performance of examiners during clinical component of the assessment (i.e. long and short cases), perceptions on clinical assessment and perceptions on overall undergraduate assessment in Surgery.

## Abbreviations

MCQ: multiple choice question
SEQ: structured essay question
OSCE: objective structured clinical examination
