# Supplementary material for: Medical undergraduates’ perceptions on the end of course assessment in Surgery in a developing country in South Asia
Source: BMC Res Notes. 2018 Oct 16;11:731. doi: 10.1186/s13104-018-3828-1 (PMC6192109; doi:10.1186/s13104-018-3828-1)
Supplement: Supplementary file 1 — Additional file 1. Questionnaire used in the survey. The questionnaire used in this study is a quantitative assessment of students’ perception on end of course assessment in Surgery. The questionnaire includes questions regarding students’ perceptions on the performance of examiners during clinical component of the assessment (i.e. long and short cases), perceptions on clinical assessment and perceptions on overall undergraduate assessment in Surgery. [file 13104_2018_3828_MOESM1_ESM.rtf]

Questionnaire			
Short cases examiners: 		Strongly	Agree	Disagree	Strongly		
		agree				disagree		
1.	


Interrupted me frequently and made me lose my train of thought 

2.	Helped me gain confidence 

3.	Made it difficult for me to know what progress I was making 

4.	Seemed keen to help me cope with a very stressful situation 

5.	Overall did a fair assessment of my clinical knowledge & skills 

Long cases examiners:					
	Strongly	Agree	Disagree	Strongly	
	agree			disagree	
1.	

Interrupted me frequently and made me lose my train of thought 

2.	Helped me gain confidence 

3.	Made it difficult for me to know what progress I was making 

4.	Seemed keen to help me cope with a very stressful situation 

5.	Overall did a fair assessment of my clinical knowledge & skills 


Clinical Assessment (Overall)	Strongly	Agree     Disagree      Strongly	
	agree	disagree	
1.	

Tasks reflected those taught 

2.	Assessment covered a wide area of knowledge and skills 

3.	Time given for assessment was adequate 

4.	The examiners were polite and professional 

5.	Assessment provided a true measure of essential clinical skills in general surgery 
6.	Assessment helped identify areas of clinical weakness and provided new opportunities to learn 


Overall assessments in Surgery
Please select the most suitable type of examination
1.	What format was the easiest? 

2.	What format would best ensure practical application of clinical knowledge? 
3.	What format would best assess whether you a good and safe doctor? 

4.	What format tests communication and counselling skills best? 
5.	What format demands organizational and time management skills the most? 
6.	What format best ensures “quality of performance” testing? 


MCQ	SEQ   Viva   OSCE     Short    Long

                                                   Cases    case
